# Using oral tegafur/uracil (UFT) plus leucovorin as adjuvant chemotherapy in stage II colorectal cancer: a propensity score matching study from Taiwan

**DOI:** 10.1186/s12885-023-11310-6

**Published:** 2023-09-25

**Authors:** Yen-Lin Yu, Wen-Ko Tseng, Chun-Kai Liao, Chien-Yuh Yeh, Hong-Hwa Chen, Yu-Hsuan Liu, Yu-Wei Liaw, Chung-Wei Fan

**Affiliations:** 1 Division of Colon and Rectal Surgery, Department of Surgery, Chang Gung Memorial Hospital, Keelung Branch, No. 222 Maijin Rd., Anle Dist., Keelung City, 20401 Taiwan; 2 Division of Colon and Rectal Surgery, Department of Surgery, Chang Gung Memorial Hospital, Linkou Branch, No. 5, Fuxing St., Guishan Dist., Taoyuan City, 33305 Taiwan; 3 Division of Colon and Rectal Surgery, Department of Surgery, Chang Gung Memorial Hospital, Kaohsiung Branch, No. 123, Dapi Rd., Niaosong Dist., Kaohsing City, 83301 Taiwan

**Keywords:** Stage II colorectal cancer, Tegafur/uracil (UFT), Adjuvant chemotherapy

## Abstract

**Background:**

Early-stage colorectal cancer had excellent outcomes after curative resection, typically. However, a perplexing survival paradox between stage II and stage III was noted. This paradox could be influenced by the administration of routine postoperative adjuvant chemotherapy and the presence of high-risk factors in stage II CRC. The objective of the study was to investigate the influence of high-risk factors on patients with stage II CRC and assess the efficacy of oral tegafur/uracil (UFT) plus leucovorin as adjuvant chemotherapy for stage II CRC patients.

**Methods:**

A retrospective study was conducted using propensity score matching at a single medical institution. A total of 1544 patients with stage II colorectal cancer who underwent radical surgery between January 2004 and January 2009 were included. The intervention used was tegafur/uracil plus leucovorin as adjuvant chemotherapy. The main outcome measures were disease-free survival and overall survival.

**Results:**

After propensity score matching, 261 patients were included in three groups: no-treatment, half-year treatment, and one-year treatment. The clinical characteristics of each group tended to be more consistent. The Cox proportional hazard models showed that tegafur/uracil treatment or not was a significant independent factor for oncological outcome. Kaplan–Meier analysis also showed significantly better disease-free survival and overall survival. Further investigation revealed that tegafur/uracil duration was an independent factor for oncological outcome. While the survival curve did not reach statistical significance, the one-year UFT treatment group demonstrated the best treatment trend.

**Conclusions:**

This study suggests that tegafur/uracil plus leucovorin is a feasible adjuvant chemotherapy regimen for patients with stage II colorectal cancer after curative surgical treatment. Prolonged tegafur/uracil plus leucovorin treatment for 12 months showed a trend towards better outcomes in patients with stage II colorectal cancer.

**Supplementary Information:**

The online version contains supplementary material available at 10.1186/s12885-023-11310-6.

## Introduction

Colorectal cancer (CRC) is the third most common cancer and second most common cause of cancer-related deaths worldwide. About 1.9 million people were newly diagnosed and 915,000 patients died of CRC in 2020 [1]. CRC has been the most common cancer and the third leading cause of cancer-related deaths in Taiwan for the past fifteen years [2]. In order to reduce the mortality of CRC, the Health Promotion Administration of the Taiwanese government started a nationwide screening program using the fecal immunochemical test (FIT) for citizens aged 50 to 75 years every two years since 2004 [3]. Under the CRC screen program, more and more individuals with early CRC were diagnosed [4].

According to the TNM staging classification, CRC that can be completely resected with no involvement of adjacent organs, lymph nodes, or distant sites is defined as early stage CRC (stage 0–III) [5, 6]. Overall, after curative resection, the treatment outcomes for early stage CRC are excellent [7]. However, analysis of patient data in the SEER (Surveillance, Epidemiology, and End Results) database showed the survival paradox between localized CRC patients (stage II) and regional CRC patients (stage III). Patients with node-negative disease and advanced colonic wall involvement (T3N0M0, stage IIa; T4aN0M0, stage IIb; and T4bN0M0, stage IIc) had worse 5-year survival than patients with limited nodal positivity and limited colonic wall involvement (T1-2N1aM0: Stage IIIa) [8].

For patients with stage III (node-positive) CRC, postoperative adjuvant chemotherapy was the accepted standard treatment in several practice guidelines either in the western or the eastern world [7, 9]. The 5-FU based adjuvant chemotherapy can improve the disease-free survival and overall survival in patients with stage III CRC [10]. For patients with stage II (node-negative) CRC, the role of postoperative adjuvant chemotherapy was still a controversial issue. Several practice guidelines have pointed out that perineural invasion, lymphovascular invasion, perforation, obstruction, poor differentiation, T4 lesion, harvesting less than 12 lymph nodes, positive margins, mucinous type, and high pre-operative carcinoembryonic antigen (CEA) levels are high-risk factors in stage II CRC and only suggested postoperative adjuvant chemotherapy in these high-risk patients with stage II CRC [9, 11, 12]. Routine postoperative adjuvant chemotherapy or not and high-risk factors were the important reasons for the survival paradox between patients with stage II and stage III CRC.

Therefore, the objective of the study was to investigate the influence of high-risk factors on patients with stage II CRC and assess the efficacy of oral tegafur/uracil (UFT) plus leucovorin as adjuvant chemotherapy for stage II CRC patients.

## Materials & methods

We collected data from patients diagnosed with node-negative CRC who underwent radical surgery between January 2004 and January 2009 at the Keelung, Linkou, and Kaohsiung branches of the Chang Gung Memorial Hospital, Taiwan. Clinical demographic data, laboratory test results, operative characteristics, pathological features, medication history, and follow-up status were obtained from patients’ electronic medical records and the Colorectal Section Tumor Registry of Chang Gung Memorial Hospital. Clinical demographic data and laboratory test results, including sex, age, carcinoembryonic antigen (CEA) level, and albumin level, were recorded. Medication history and follow-up status, such as the use and duration of adjuvant chemotherapy, disease-free survival (DFS), and overall survival (OS) were collected. The operative characteristics and pathological features were recorded, including tumor location, perforation, obstruction, perineural invasion, lymphovascular invasion, histological differentiation, TNM stage, and number of harvested lymph nodes. DFS was defined as the interval from initial surgical intervention to the date of first recurrence, death, or last follow-up. OS was defined as the interval from initial surgical intervention to the date of death or last follow-up. All patients were continuously followed-up until 2015 or death. The median follow-up period was 72.2 months. Data collection and analysis were supervised and approved by the Institutional Review Board of Chang Gung Memorial Hospital in Taiwan (IRB No. 202201583B0).

**Fig. 1:**
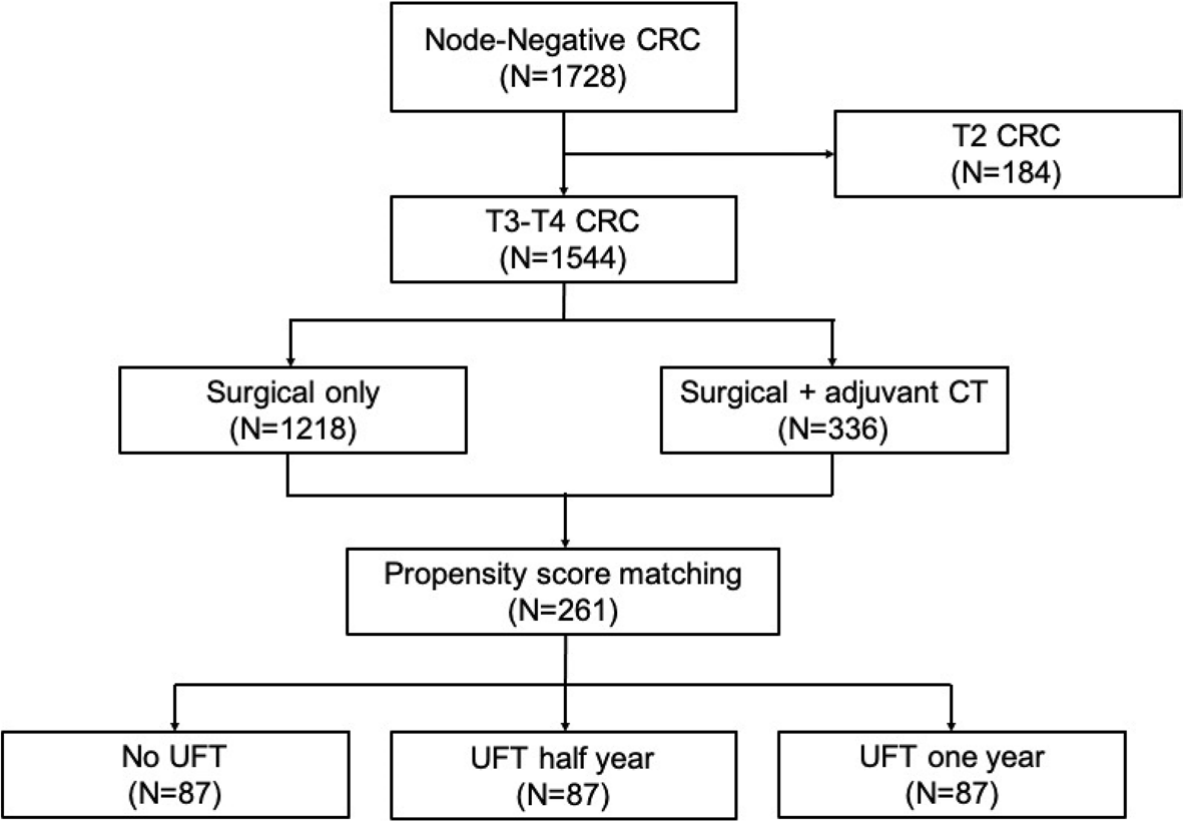
The flowchart of patient collection and screening in this study

After surgery, patients with pathology confirmed T1 or T2 colorectal cancer (stage I) were excluded from the study. A total of 1544 stage II cancer patients were enrolled in the analysis. Of the enrolled patients, 1218 underwent surgical intervention and 336 underwent surgery intervention and adjuvant chemotherapy treatment. The UFT was chosen as the adjuvant chemotherapy regimen and administered at 400 mg as tegafur orally in two divided doses after meals for 3 weeks, followed by 1 week rest. The treatment was repeated for half or one year depending on patients’ performance and surgeons’ experience. Considering selection bias in non-randomized studies and achieving balanced covariates across treatment groups, propensity score matching (PSM) was performed using a logistic regression model, with the use and period of the oral form of adjuvant chemotherapy set as dependent variables. Patients in all groups were matched according to their clinical and pathological characteristics, including perineural invasion, lymphovascular invasion, perforation, obstruction, poor differentiation, T4 lesion, harvesting less than 12 lymph nodes, and tumor location. A1:1:1 PSM matching was performed with the nearest neighbor matching method using calipers of width equal to 0.2 in no UFT treatment, UFT treatment for half year, and UFT treatment for one year. A selection flowchart is shown in Fig. 1.

Statistical analyses were performed using SAS software (version 9.4; SAS Institute, Inc.; Cary, NC, USA) and the Statistical Package for the Social Sciences version 26 (IBM Corp., Armonk, NY, USA). Clinical and pathological characteristics were compared using the chi-squared test for categorical variables. PSM was performed by using a logistic regression model. After matching, the univariate Cox proportional hazards model was applied to evaluate the relationship between clinical characteristics, DFS, and OS. The statistically significant variables identified in the univariate analysis were applied to the multivariate Cox proportional hazard model to identify the independent variables that might affect DFS and OS. Data on the duration of DFS and OS were plotted using the Kaplan–Meier method. DFS and OS curves were compared using log-rank tests. All statistical differences were considered statistically significant at *P* value < 0.05.

## Results

### Propensity score matching

We enrolled and analyzed 1544 patients with stage II CRC who underwent radical surgical intervention and divided them into a no UFT treatment group, half-year UFT treatment group, and one-year UFT treatment group. Table 1 shows the clinical characteristics of the patients before and after matching. The distribution of clinical characteristics of patients with stage II CRC in each treatment group before the matching was uneven. Significant differences in each treatment group were observed in age, sex, high-risk (perineural invasion, lymphovascular invasion, perforation, poor differentiation, T4 lesion, harvesting < 12 lymph nodes), tumor location (rectum), CEA level, and nutritional status (albumin level). After propensity score matching, 87 patients were included in each treatment group. The distribution of the clinical characteristics of patients with stage II CRC in each treatment group tended to be more consistent. Except for older age, harvesting less than 12 lymph nodes and rectal tumors were higher in the no UFT treatment group; the distribution of the remaining clinical characteristics was similar in between treatment groups.

**Table 1:** The clinical characteristics of patients with stage II CRC before and after propensity score matching

	**Before PSM**					**After PSM**				
**Clinical characteristics**	**Total**	**No UFT**	**UFT** **half year**	**UFT** **one year**	***P* value**	**Total**	**No UFT**	**UFT** **half year**	**UFT** **one year**	***P* value**
Patient number (%)	1554(100)	1218(78.3)	148(9.5)	188(12.0)		261(100)	87(33.3)	87(33.3)	87(33.3)	
Age > 65	968(53.6)	803(66.0)	68(45.9)	97(51.6)	< 0.001	140(53.6)	57(65.5)	39(44.8)	44(50.6)	0.018
Male	890(57.3)	687(56.4)	79(53.4)	124(66.0)	0.029	155(59.4)	48(55.2)	50(57.5)	57(65.5)	0.345
Perineural invasion	234(15.1)	165(13.5)	33(22.3)	36(19.1)	0.005	71(27.2)	25(28.7)	24(27.6)	22(25.3)	0.873
Lymphvascular invasion	132(8.5)	73(6.0)	18(12.2)	41(21.8)	< 0.001	58(22.2)	21(24.1)	16(18.4)	21(24.1)	0.575
Perforation	58(3.7)	40(3.3)	12(8.1)	6(3.2)	0.013	20(7.7)	6(6.9)	10(11.5)	4(4.6)	0.220
Obstruction	209(13.5)	158(13.0)	19(12.8)	32(17.0)	0.313	46(17.6)	15(17.2)	15(17.2)	16(18.4)	0.974
Poor differentiation	86(5.5)	61(5.0)	18(12.2)	7(3.7)	0.001	14(5.4)	5(5.7)	3(3.4)	6(6.9)	0.590
T4 lesion	553(35.6)	414(34.0)	38(25.7)	101(53.7)	< 0.001	89(34.1)	27(31.0)	33(37.9)	29(33.3)	0.620
LN < 12	427(27.5)	335(27.5)	15(10.1)	77(41.0)	< 0.001	54(20.7)	27(31.0)	15(17.2)	12(13.8)	0.012
High risk	1040(66.9)	775(63.6)	93(62.8)	172(91.5)	< 0.001	209(80.1)	65(74.7)	72(82.8)	72(82.8)	0.308
Right colon	453(29.2)	348(28.6)	53(35.8)	52(27.7)	0.167	72(27.6)	21(24.1)	21(24.1)	30(34.5)	0.211
Rectum	563(36.2)	460(37.8)	39(26.4)	64(34.0)	0.019	110(42.1)	53(60.9)	37(42.5)	20(23.0)	< 0.001
CEA > 5(ng/mL)	499(33.5)	384(33.0)	63(43.2)	52(28.7)	0.017	96(37.5)	35(40.2)	36(42.4)	25(29.8)	0.194
Albumin < 3.5(g/dL)	426(28.4)	324(27.8)	35(24.3)	67(35.8)	0.039	72(28.3)	20(24.4)	20(23.5)	32(36.8)	0.098

*P* value was determined by chi-square test for multiple comparisons

### Effect of UFT treatment on patients with stage II CRC

**Fig. 2:**
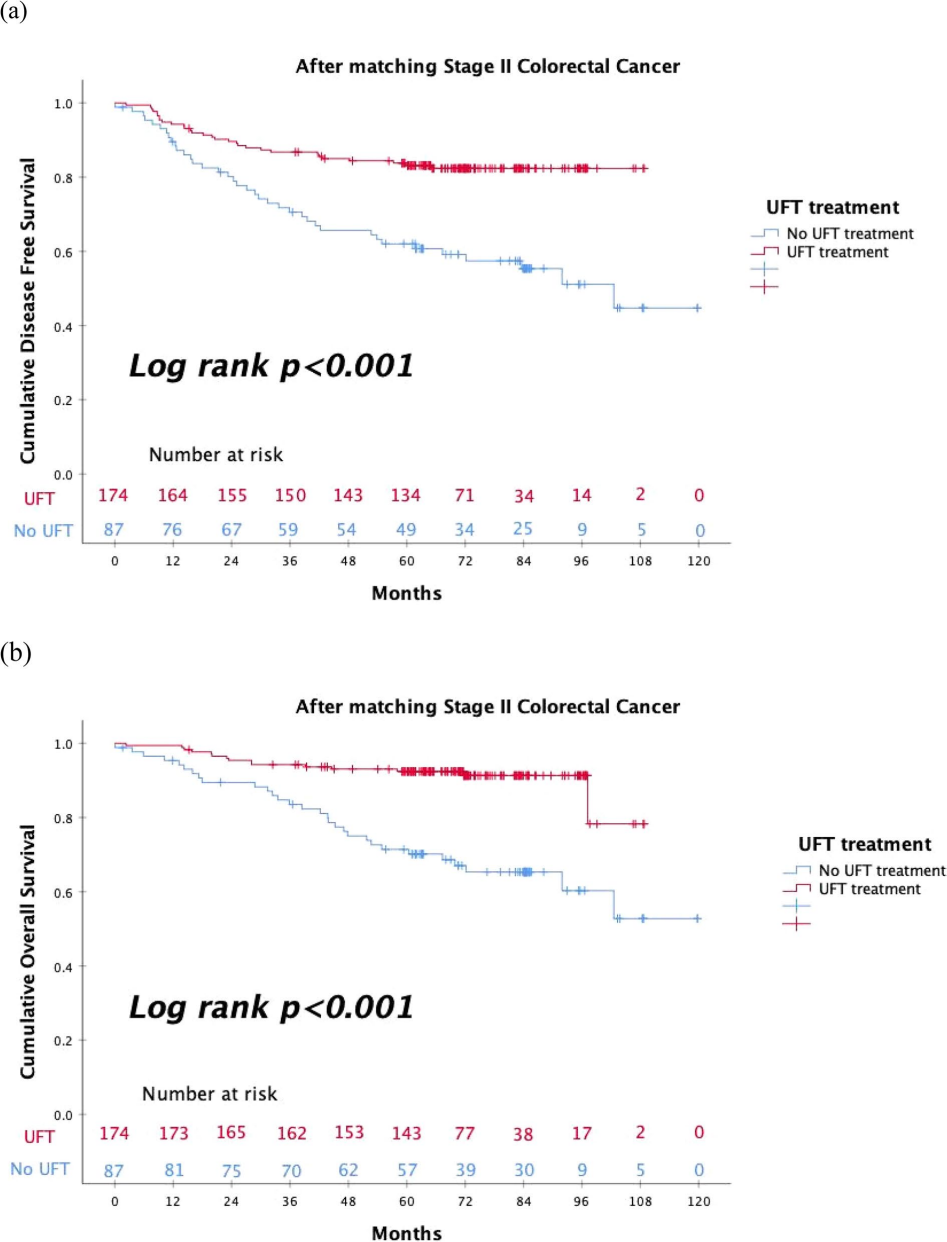
**a** The DFS analysis according to UFT treatment in patients with stage II CRC. **b** The OS analysis according to UFT treatment in patients with stage II CRC

After matching, under the equal distribution of the clinical characteristics across treatment groups, univariate and multivariate Cox proportional hazard models were used to explore the factors that affect DFS and OS. We found that perineural invasion, tumor location, and UFT treatment were significant independent factors for DFS (Table 2). Similarly, perineural invasion, perforation, tumor location, and UFT treatment were significant independent factors for OS (Table 2). We also performed Kaplan–Meier analysis to determine the DSF and OS curves according to the UFT treatment. And the Patients with stage II CRC in the UFT treatment group displayed significantly better DFS (log-rank test: *p* < 0.001) and OS (log-rank test: *p* < 0.001) than those in the no UFT treatment group (Fig. 2a and b). The same UFT treatment efficacy was also obtained when we analyzed colon cancer (log-rank test: *p* < 0.001) and rectal cancer (log-rank test: *p* = 0.015) separately (Supplementary Fig. 1a and b).

**Table 2:** The univariate and multivariate analysis of the prognostic factors for disease-free survival and overall survival according to UFT treatment using Cox proportional hazard model

	**Disease-free survival**				**Overall survival**			
**Variables**	**Univariate Cox Regression**		**Multivariate Cox Regression**		**Univariate Cox Regression**		**Multivariate Cox Regression**	
	HR (95% CI)	*P* value	HR (95% CI)	*P* value	HR (95% CI)	*P* value	HR (95% CI)	*P* value
Age ≥ 65	1.246(0.770–2.016)	0.370			2.157(1.156–4.092)	0.016	1.436(0.739–2.793)	0.286
Sex	0.924(0.571–1.494)	0.746			1.296(0.703–2.389)	0.405		
Perineural invasion	2.347(1.452–3.794)	< 0.001	2.379(1.432–3.953)	0.001	3.114(1.732–5.597)	< 0.001	2.835(1.511–5.317)	0.001
Lymphovascular invasion	1.202(0.694–2.081)	0.512			1.258(.650–2.437)	0.496		
Perforation	1.958(0.935–4.102)	0.075	2.219(0.927–5.312)	0.073	3.359(1.554–7.275)	0.002	5.481(2.195–13.68)	< 0.001
Obstruction	1.166(0.635–2.139)	0.621			1.162(0.556–2.432)	0.689		
Poor differentiation	0.985(0.359–2.706)	0.977			0.768(0.186–3.172)	0.715		
T4 lesion	1.327(0.814–2.163)	0.257			0.933(0.498–1.749)	0.830		
LN < 12	1.882(1.116–3.174)	0.018	1.290(0.726–2.295)	0.385	2.844(1.555–5.203)	0.001	1.286(0.632–2.618)	0.487
High risk	1.368(0.717–2.613)	0.342			1.679(0.708–3.980)	0.239		
CEA > 5(ng/mL)	1.716(1.057–2.785)	0.029	1.472(0.899–2.408)	0.124	1.481(0.811–2.707)	0.201		
Albumin < 3.5(g/dL)	1.459(0.879–2.421)	0.144			1.674(0.901–3.110)	0.103		
Right vs. Left	1.492(0.834–2.667)	0.177			1.527(0.741–3.149)	0.251		
Colon vs. Rectum	1.941(1.199–3.141)	0.007	1.818(1.045–3.364)	0.034	2.291(1.253–4.191)	0.007	2.411(1.212–4.799)	0.012
Radiation treatment	1.310(0.464–3.697)	0.610			0.770(0.182–3.262)	0.723		
UFT treatment	0.352(0.218–0.569)	< 0.001	0.429(0.252–0.731)	0.003	0.233(0.125–0.434)	< 0.001	0.321(0.163–0.630)	0.001

*P* value was determined by Cox regression for univariate and multivariate analyses

### UFT treatment duration in patients with stage II CRC

**Fig. 3:**
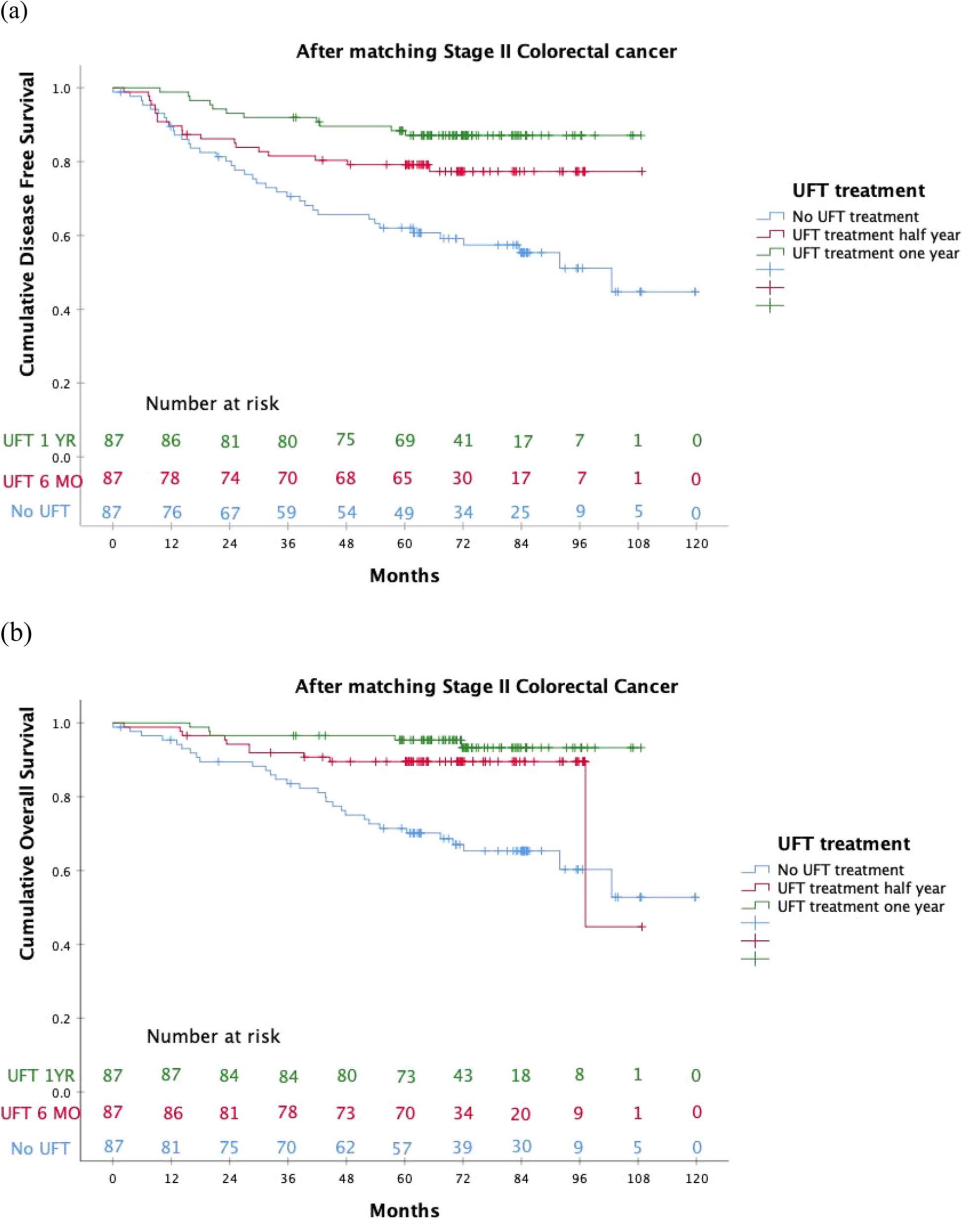
**a** The DFS analysis according to UFT treatment duration in patients with stage II CRC. **b** The OS analysis according to UFT treatment duration in patients with stage II CRC

Further analysis revealed that perforation, UFT half-year treatment, and UFT one-year treatment were significant independent factors for DFS in univariate and multivariate Cox proportional hazard models (Table 3). Perineural invasion, perforation, tumor location, UFT half-year treatment, and UFT one-year treatment were also significant independent factors for OS in patients with stage II CRC (Table 3). Although the Kaplan–Meier analysis to determine the DSF and OS curves according to UFT treatment duration did not reach statistical significance (log-rank test of DFS: *p* = 0.084 and log-rank test of OS: *p* = 0.132), the best treatment trend was still observed in the one-year UFT treatment group (Fig. 3a and b).

**Table 3:** The univariate and multivariate analysis of the prognostic factors for disease-free survival and overall survival according to UFT treatment duration using Cox proportional hazard model

	**Disease-free survival**				**Overall survival**			
**Variables**	**Univariate Cox Regression**		**Multivariate Cox Regression**		**Univariate Cox Regression**		**Multivariate Cox Regression**	
	HR (95% CI)	*P* value	HR (95% CI)	*P* value	HR (95% CI)	*P* value	HR (95% CI)	*P* value
Age ≥ 65	1.246(0.770–2.016)	0.370			2.157(1.156–4.092)	0.016	1.461(0.751–2.842)	0.264
Sex	0.924(0.571–1.494)	0.746			1.296(0.703–2.389)	0.405		
Perineural invasion	2.347(1.452–3.794)	< 0.001	2.372(1.426–3.945)	0.001	3.114(1.732–5.597)	< 0.001	2.844(1.514–5.342)	0.001
Lymphovascular invasion	1.202(0.694–2.081)	0.512			1.258(.650–2.437)	0.496		
Perforation	1.958(0.935–4.102)	0.075	2.083(0.867–5.006)	0.101	3.359(1.554–7.275)	0.002	5.197(2.069–13.05)	< 0.001
Obstruction	1.166(0.635–2.139)	0.621			1.162(0.556–2.432)	0.689		
Poor differentiation	0.985(0.359–2.706)	0.977			0.768(0.186–3.172)	0.715		
T4 lesion	1.327(0.814–2.163)	0.257			0.933(0.498–1.749)	0.830		
LN < 12	1.882(1.116–3.174)	0.018	1.284(0.721–2.287)	0.395	2.844(1.555–5.203)	0.001	1.263(0.620–2.575)	0.520
High risk	1.368(0.717–2.613)	0.342			1.679(0.708–3.980)	0.239		
CEA > 5(ng/mL)	1.716(1.057–2.785)	0.029	1.441(0.879–2.364)	0.148	1.481(0.811–2.707)	0.201		
Albumin < 3.5(g/dL)	1.459(0.879–2.421)	0.144			1.674(0.901–3.110)	0.103		
Right vs. Left	1.492(0.834–2.667)	0.177			1.527(0.741–3.149)	0.251		
Colon vs. Rectum	1.941(1.199–3.141)	0.007	1.728(0.992–3.010)	0.054	2.291(1.253–4.191)	0.007	2.287(1.145–4.570)	0.019
Radiation treatment	1.310(0.464–3.697)	0.610			0.770(0.182–3.262)	0.723		
UFT half year	0.469(0.270–0.816)	0.007	0.516(0.286–0.930)	0.028	0.323(0.157–0.663)	0.002	0.379(0.178–0.810)	0.012
UFT one year	0.246(0.125–0.481)	< 0.001	0.318(0.151–0.672)	0.003	0.150(0.058–0.387)	< 0.001	0.240(0.088–0.656)	0.005

*P* value was determined by Cox regression for univariate and multivariate analyses

## Discussion

This retrospective cohort study included a large sample size and real-world experiences from our institutes. After balancing the multiple high-risk factors that may affect oncological prognosis, our results demonstrate that patients with stage II CRC who received oral UFT plus leucovorin as postoperative adjuvant chemotherapy had better DFS and OS. We also noted that a prolonged UFT treatment period of 12 months had a trend towards better DFS and OS in patients with stage II CRC.

Several global practice guidelines have indicated that radical surgical resection is the main curative treatment for locoregional (stage I to III) CRC. Radical resection involves complete removal of the tumor and the associated major lymphovascular pedicles of the affected colonic segment [7, 12, 13]. In theory, locoregional (stage I to III) CRC can potentially be a candidate for curative R0 resection. However, cancer recurrence occurs through micrometastases that cannot be detected before and during surgery. The goal of postoperative adjuvant chemotherapy is to eradicate micrometastases, prevent recurrence, and improve prognosis after curative R0 resection. For patients with stage III CRC, postoperative adjuvant chemotherapy is a standard treatment [9]. Adjuvant chemotherapy can decrease disease recurrence by approximately 30% and the mortality rate in patients with stage III CRC [14]. However, the issue of postoperative adjuvant chemotherapy for patients with stage II CRC has been controversial. There is also no consensus regarding which regimen should be used for patients with stage II CRC.

**Fig. 4:**
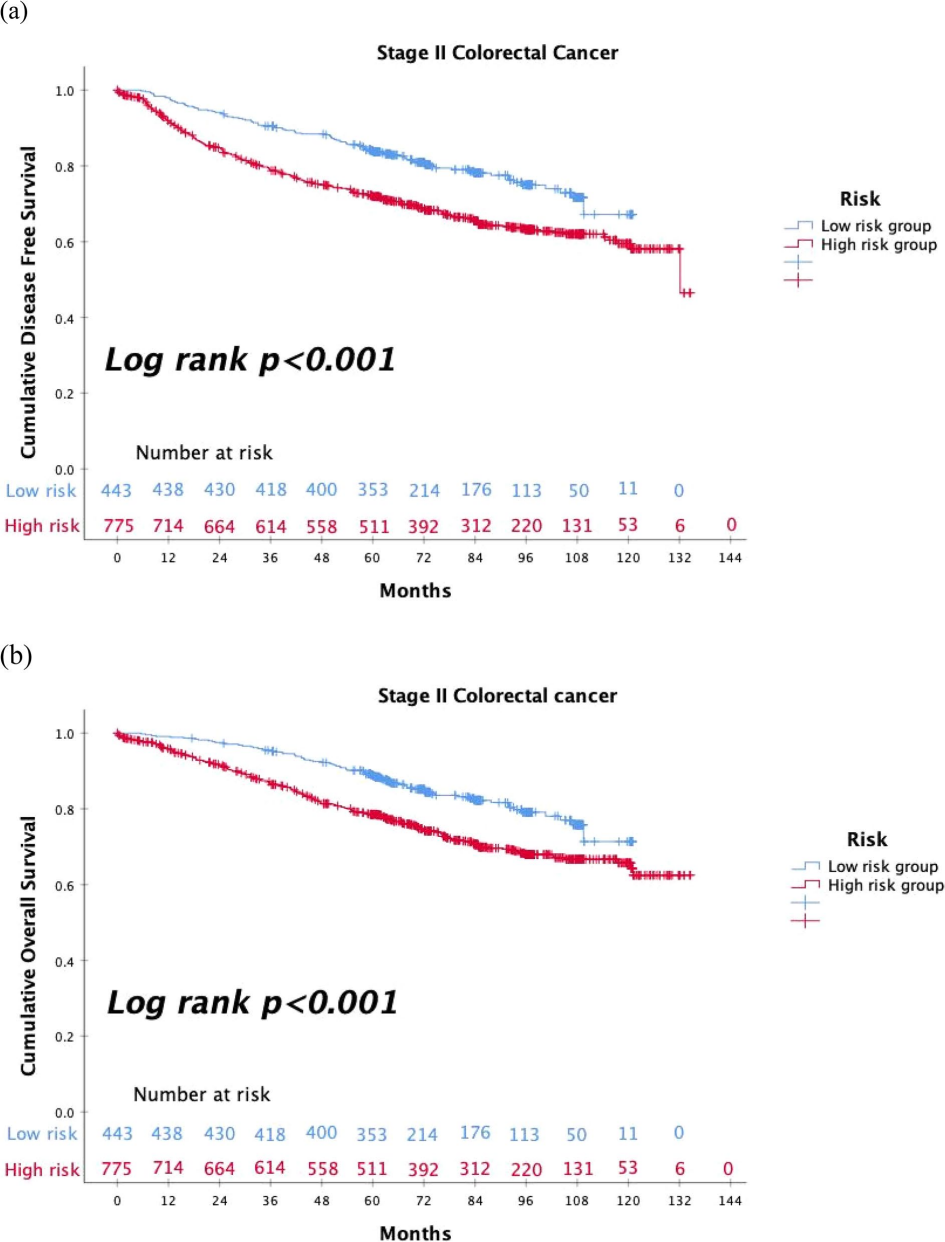
**a** The DFS analysis of low-risk and high-risk groups of patients with stage II CRC. **b** The OS analysis of low-risk and high-risk groups of patients with stage II CRC

In stage II CRC patients, the range of 5-year survival rates widely varies from 89.6% to 55.0% in several substages condition [15]. Many practice guidelines divide patients with stage II CRC into high-risk and low-risk groups according to different clinical and pathological characteristics, including perforation, obstruction, perineural invasion, lymphovascular invasion, poor differentiation, T4 lesions, harvesting less than 12 lymph nodes, positive margins, mucinous type, and high preoperative CEA levels. And postoperative adjuvant chemotherapy is recommended for high-risk groups with different regimens and durations [9, 11, 12]. However, the definitions of risk factors in these guidelines are still slightly different. The National Comprehensive Cancer Network (NCCN) guideline regards close, indeterminate, and positive resection margins as a special risk factor [9]. However, different from other guidelines those of the American Society of Clinical Oncology (ASCO) guidelines consider mucinous tumors as a risk factor [11]. In the European Society for Medical Oncology (ESMO) guidelines for localized colon cancer, the T4 lesion including perforation is considered the most critical risk factor, and high preoperative CEA levels are also seen as a special risk factor [12]. The risk factors mentioned in all three guidelines are regarded as the consensus of risk factors in our institutes, including perineural invasion, lymphovascular invasion, perforation, obstruction, poor differentiation, T4 lesion, and harvesting less than 12 lymph nodes. Our database also revealed that stage II CRC patients with high-risk factors had poor prognoses for DFS and OS (Fig. 4a and b). In this study, propensity score matching enabled even distribution of the above clinical characteristics between treatment groups that may affect the oncological prognosis. Thereby, we can objectively evaluate the effect of UFT plus leucovorin as adjuvant chemotherapy in patients with stage II CRC.

Tegafur/uracil (UFT or UFUR) is a prescription medication released in Japan during the 1980s. It is a 1:4 molar ratio of tegafur and uracil. Tegafur is a precursor of 5‐fluorouracil (5-FU), which can be absorbed by the gastrointestinal tract and metabolized to active 5-FU by cytochrome P450 enzymes in the liver. Uracil is a competitive inhibitor of dihydropyrimidine dehydrogenase (DPD) and can reduce 5‐FU into fluorodihydrouracil (FDHU) [16]. And leucovorin can increase thymidylate synthase (TS) inhibition, modulate the cellular cytotoxicity of 5-FU, and potentiate its tumor control [17, 18]. UFT has been widely used in Asia and Europe for several types of cancer, including breast, [19] lung, [20] gastric, [21] and head and neck cancers [22]. In addition, UFT has also been used in different stages of CRC as adjuvant or palliative treatment [23–26].

A randomized controlled trial by Chu Matsuda et al. did not demonstrate the superiority of 1-year postoperative adjuvant UFT treatment over surgery alone in stage II colon cancer [27]. However, according to a publication from this clinical trial, the recurrence rate was lower in the UFT treatment group than in the surgical alone group (10.4% vs. 13.4%), and improvement in relapse-free survival for the UFT group, although statistically insignificant, was also noted. The adherence rate to 1-year UFT treatment was only 60.8% in this study because of adverse events and financial factors. According to previous treatment experience, the adherence rate to a complete planned schedule may affect the benefit of chemotherapy in CRC patients [28, 29]. Compared to the JFMC46-1201 clinical trial in Japan, when the adherence rate to half-year UFT treatment was 71.8%, significant survival benefits for patients with stage II high-risk colon cancer were observed [30]. In our study, we collected a large number of real-world data from our institute. After propensity score matching, under the 100% adherence rate in each treatment arm, that the administration of UFT plus leucovorin as adjuvant chemotherapy in patients with stage II CRC offered survival benefits.

It remains unclear why long-term postoperative adjuvant chemotherapy achieves better DFS and OS in patients with stage II CRC. Compared to conventional chemotherapy, metronomic chemotherapy is another treatment concept for the application of anticancer drugs. This long-term, low-dose, and no-rest period chemotherapy modality was first introduced by Hanahan et al. in 2000 [31]. Conventional chemotherapy uses individual maximum tolerated doses to achieve cytotoxicity in cancer cells. In contrast, metronomic chemotherapy administers lower maximum doses but more frequently to achieve the goal of anti-angiogenesis and immunomodulation to affect the tumor microenvironment [32, 33]. Under the effect of anti-angiogenesis and immunomodulation, long-term tumor dormancy would also be induced by metronomic chemotherapy [34]. Metronomic chemotherapy is often administered in oral form, which is more convenient for patients and has lower medical cost than conventional intravenous chemotherapy. Because of the different mechanisms of anticancer effects, metronomic chemotherapy affects endothelial cells and immune cells in the tumor microenvironment and does not induce severe side effects or drug resistance [35]. Under the concept of metronomic chemotherapy, which is mentioned above, the oral form of UFT plus leucovorin as adjuvant chemotherapy is a suitable choice for patients with CRC [36]. And this may explain the benefit of DFS and OS in patients with stage II CRC who received oral UFT plus leucovorin after curative radical surgery in our study.

Our study has some limitations. First, it was not a prospective randomized control trial, and selection bias may have existed in our primary retrospective cohort between January 2004 and January 2009. Second, adjuvant chemotherapy with UFT plus leucovorin and the treatment period might depend on the patients’ performance and surgeons’ experience. We attempted to reduce these biases as much as possible using PSM. Third, several molecular reviews for microsatellite instability (MSI) testing [37], RAS and BRAF mutations, pathological reports for tumor budding, [9] and desmoplastic reaction, and liquid biopsy with circulating tumor DNA detection [38] were not routine requirements in our primary cohort between January 2004 and January 2009. In the future, multiple randomized controlled trials and meta-analyses mut be performed to evaluate the effect of adjuvant chemotherapy with UFT plus leucovorin in patients with stage II CRC.

## Conclusion

During the COVID-19 pandemic, several topics in cancer care have changed. Minimally visiting hospitals, appropriately allocating medical resources, and effective cancer treatment are the most important topics of this generation. Oral adjuvant chemotherapy is an acceptable treatment for stage II CRC patients [39]. Our study demonstrates that UFT plus leucovorin is a feasible adjuvant chemotherapy regimen for patients with stage II CRC after curative surgical treatment. Because of the meager incidence rate of side effects, prolonged UFT plus leucovorin treatment for 12 months showed a trend towards better DFS and OS in patients with stage II CRC.

### Supplementary Information

**Additional file 1:** **Supplementary Fig. 1.** (a) The OS analysis according to UFT treatment in patients with stage II colon cancer. (b) The OS analysis according to UFT treatment in patients with stage II rectal cancer.

## Data Availability

The datasets used and analyzed during the current study are available from the corresponding author on reasonable request.

## Abbreviations

CRC: Colorectal cancer
FIT: Fecal immunochemical test
SEER: Surveillance, epidemiology, and end results
UFT: Tegafur/uracil
PSM: Propensity score matching
CEA: Carcinoembryonic antigen
Hb: Hemoglobin
DFS: Disease-free survival
OS: Overall survival
RCT: Randomized controlled trial

## References

[1] Sung H, Ferlay J, Siegel RL, Laversanne M, Soerjomataram I, Jemal A, Bray F (2021). Global cancer statistics 2020: GLOBOCAN estimates of incidence and mortality worldwide for 36 cancers in 185 countries. CA Cancer J Clin.

[2] Health Promotion Administration MoHaW (2022). Cancer Registry Annual Report, 2019 Taiwan. Taiwan: Health Promotion Administration, Ministry of Health and Welfare.

[3] Wang YW, Chen HH, Wu MS, Chiu HM (2018). Current status and future challenge of population-based organized colorectal cancer screening: Lesson from the first decade of Taiwanese program. J Formos Med Assoc.

[4] Kubisch CH, Crispin A, Mansmann U, Goke B, Kolligs FT (2016). Screening for colorectal cancer is associated with lower disease stage: a population-based study. Clin Gastroenterol Hepatol.

[5] Freeman HJ (2013). Early stage colon cancer. World J Gastroenterol.

[6] Costas-Chavarri A, Nandakumar G, Temin S, Lopes G, Cervantes A, Cruz Correa M, Engineer R, Hamashima C, Ho GF, Huitzil FD (2019). Treatment of patients with early-stage colorectal cancer: ASCO resource-stratified guideline. J Glob Oncol.

[7] Hashiguchi Y, Muro K, Saito Y, Ito Y, Ajioka Y, Hamaguchi T, Hasegawa K, Hotta K, Ishida H, Ishiguro M (2020). Japanese Society for Cancer of the Colon and Rectum (JSCCR) guidelines 2019 for the treatment of colorectal cancer. Int J Clin Oncol.

[8] Abdel-Rahman O (2018). Revisiting Dukes' paradigm; some node positive colon cancer patients have better prognosis than some node negative patients. Clin Transl Oncol.

[9] NCCN Clinical Practice Guidelines in Oncology (NCCN Guidelines®) Colon cancer (Version 2.2021). https://www.nccn.org/professionals/physician_gls/pdf/colon.pdf.

[10] André T, de Gramont A, Vernerey D, Chibaudel B, Bonnetain F, Tijeras-Raballand A, Scriva A, Hickish T, Tabernero J, Van Laethem JL (2015). Adjuvant fluorouracil, leucovorin, and oxaliplatin in stage ii to iii colon cancer: updated 10-year survival and outcomes according to BRAF mutation and mismatch repair status of the mosaic study. J Clin Oncol.

[11] Baxter NN, Kennedy EB, Bergsland E, Berlin J, George TJ, Gill S, Gold PJ, Hantel A, Jones L, Lieu C (2022). Adjuvant therapy for stage ii colon cancer: ASCO guideline update. J Clin Oncol.

[12] Argilés G, Tabernero J, Labianca R, Hochhauser D, Salazar R, Iveson T, Laurent-Puig P, Quirke P, Yoshino T, Taieb J (2020). Localised colon cancer: ESMO clinical practice guidelines for diagnosis, treatment and follow-up. Ann Oncol.

[13] Vogel JD, Felder SI, Bhama AR, Hawkins AT, Langenfeld SJ, Shaffer VO, Thorsen AJ, Weiser MR, Chang GJ, Lightner AL (2022). The american society of colon and rectal surgeons clinical practice guidelines for the management of colon cancer. Dis Colon Rectum.

[14] Sargent D, Sobrero A, Grothey A, O'Connell MJ, Buyse M, Andre T, Zheng Y, Green E, Labianca R, O'Callaghan C (2009). Evidence for cure by adjuvant therapy in colon cancer: observations based on individual patient data from 20,898 patients on 18 randomized trials. J Clin Oncol.

[15] Crooke H, Kobayashi M, Mitchell B, Nwokeji E, Laurie M, Kamble S, McKenna M, Masood A, Korytowsky B (2018). Estimating 1- and 5-year relative survival trends in colorectal cancer (CRC) in the United States: 2004 to 2014. J Clin Oncol.

[16] García-Alfonso P, Muñoz Martín AJ, Ortega Morán L, Soto Alsar J, Torres Pérez-Solero G, Blanco Codesido M, CalvoFerrandiz PA, Grasso Cicala S (2021). Oral drugs in the treatment of metastatic colorectal cancer. Ther Adv Med Oncol.

[17] Thirion P, Michiels S, Pignon JP, Buyse M, Braud AC, Carlson RW, O'Connell M, Sargent P, Piedbois P (2004). Modulation of fluorouracil by leucovorin in patients with advanced colorectal cancer: an updated meta-analysis. J Clin Oncol.

[18] Grogan L, Sotos GA, Allegra CJ (1993). Leucovorin modulation of fluorouracil. Oncology (Williston Park).

[19] Nakayama T, Noguchi S (2010). Therapeutic usefulness of postoperative adjuvant chemotherapy with Tegafur-Uracil (UFT) in patients with breast cancer: focus on the results of clinical studies in Japan. Oncologist.

[20] Kato H, Ichinose Y, Ohta M, Hata E, Tsubota N, Tada H, Watanabe Y, Wada H, Tsuboi M, Hamajima N (2004). A randomized trial of adjuvant chemotherapy with uracil-tegafur for adenocarcinoma of the lung. N Engl J Med.

[21] Aykan NF, Idelevich E (2008). The role of UFT in advanced gastric cancer. Ann Oncol.

[22] Fesneau M, Pointreau Y, Chapet S, Martin L, Pommier P, Alfonsi M, Laguerre B, Feham N, Berger C, Garaud P (2010). Concomitant chemoradiotherapy using carboplatin, tegafur-uracil and leucovorin for stage III and IV head-and-neck cancer: results of GORTEC Phase II study. Int J Radiat Oncol Biol Phys.

[23] Hasegawa K, Saiura A, Takayama T, Miyagawa S, Yamamoto J, Ijichi M, Teruya M, Yoshimi F, Kawasaki S, Koyama H (2016). Adjuvant Oral Uracil-Tegafur with Leucovorin for Colorectal Cancer Liver Metastases: A Randomized Controlled Trial. PLoS ONE.

[24] Shimada Y, Hamaguchi T, Mizusawa J, Saito N, Kanemitsu Y, Takiguchi N, Ohue M, Kato T, Takii Y, Sato T (2014). Randomised phase III trial of adjuvant chemotherapy with oral uracil and tegafur plus leucovorin versus intravenous fluorouracil and levofolinate in patients with stage III colorectal cancer who have undergone Japanese D2/D3 lymph node dissection: final results of JCOG0205. Eur J Cancer.

[25] Lembersky BC, Wieand HS, Petrelli NJ, O'Connell MJ, Colangelo LH, Smith RE, Seay TE, Giguere JK, Marshall ME, Jacobs AD (2006). Oral uracil and tegafur plus leucovorin compared with intravenous fluorouracil and leucovorin in stage II and III carcinoma of the colon: results from National Surgical Adjuvant Breast and Bowel Project Protocol C-06. J Clin Oncol.

[26] Miyake Y, Nishimura J, Kato T, Ikeda M, Tsujie M, Hata T, Takemasa I, Mizushima T, Yamamoto H, Sekimoto M (2018). Phase III trial comparing UFT + PSK to UFT + LV in stage IIB, III colorectal cancer (MCSGO-CCTG). Surg Today.

[27] Matsuda C, Ishiguro M, Teramukai S, Kajiwara Y, Fujii S, Kinugasa Y, Nakamoto Y, Kotake M, Sakamoto Y, Kurachi K (2018). A randomised-controlled trial of 1-year adjuvant chemotherapy with oral tegafur-uracil versus surgery alone in stage II colon cancer: SACURA trial. Eur J Cancer.

[28] Ahmed S, Ahmad I, Zhu T, Arnold FP, Faiz Anan G, Sami A, Yadav SK, Alvi R, Haider K (2010). Early discontinuation but not the timing of adjuvant therapy affects survival of patients with high-risk colorectal cancer: a population-based study. Dis Colon Rectum.

[29] Antonio M, Carmona-Bayonas A, Saldaña J, Navarro V, Tebé C, Salazar R, Borràs JM (2018). Factors Predicting Adherence to a Tailored-Dose Adjuvant Treatment on the Basis of Geriatric Assessment in Elderly People With Colorectal Cancer: A Prospective Study. Clin Colorectal Cancer.

[30] Sadahiro S, Sakamoto K, Tsuchiya T, Takahashi T, Ohge H, Sato T, Kondo K, Ogata Y, Baba H, Itabashi M (2022). Prospective observational study of the efficacy of oral uracil and tegafur plus leucovorin for stage II colon cancer with risk factors for recurrence using propensity score matching (JFMC46–1201). BMC Cancer.

[31] Hanahan D, Bergers G, Bergsland E (2000). Less is more, regularly: metronomic dosing of cytotoxic drugs can target tumor angiogenesis in mice. J Clin Invest.

[32] Chen YL, Chang MC, Cheng WF (2017). Metronomic chemotherapy and immunotherapy in cancer treatment. Cancer Lett.

[33] Biziota E, Mavroeidis L, Hatzimichael E, Pappas P (2017). Metronomic chemotherapy: A potent macerator of cancer by inducing angiogenesis suppression and antitumor immune activation. Cancer Lett.

[34] Páez D, Labonte MJ, Bohanes P, Zhang W, Benhanim L, Ning Y, Wakatsuki T, Loupakis F, Lenz HJ (2012). Cancer dormancy: a model of early dissemination and late cancer recurrence. Clin Cancer Res.

[35] Gnoni A, Silvestris N, Licchetta A, Santini D, Scartozzi M, Ria R, Pisconti S, Petrelli F, Vacca A, Lorusso V (2015). Metronomic chemotherapy from rationale to clinical studies: a dream or reality?. Crit Rev Oncol Hematol.

[36] Huang WY, Ho CL, Lee CC, Hsiao CW, Wu CC, Jao SW, Yang JF, Lo CH, Chen JH (2017). Oral tegafur-uracil as metronomic therapy following intravenous FOLFOX for stage III colon cancer. PLoS ONE.

[37] Eriksson J, Amonkar M, Al-Jassar G, Lambert J, Malmenäs M, Chase M, Sun L, Kollmar L, Vichnin M (2019). Mismatch repair/microsatellite instability testing practices among us physicians treating patients with advanced/metastatic colorectal cancer. J Clin Med.

[38] Osumi H, Shinozaki E, Yamaguchi K, Zembutsu H (2019). Clinical utility of circulating tumor DNA for colorectal cancer. Cancer Sci.

[39] Vecchione L, Stintzing S, Pentheroudakis G, Douillard JY, Lordick F (2020). ESMO management and treatment adapted recommendations in the COVID-19 era: colorectal cancer. ESMO Open.

