# Supplementary material for: Using oral tegafur/uracil (UFT) plus leucovorin as adjuvant chemotherapy in stage II colorectal cancer: a propensity score matching study from Taiwan
Source: BMC Cancer. 2023 Sep 25;23:900. doi: 10.1186/s12885-023-11310-6 (PMC10518963; doi:10.1186/s12885-023-11310-6)

Supplementary Figure 1:

(a) The OS analysis according to UFT treatment in patients with stage II colon cancer.


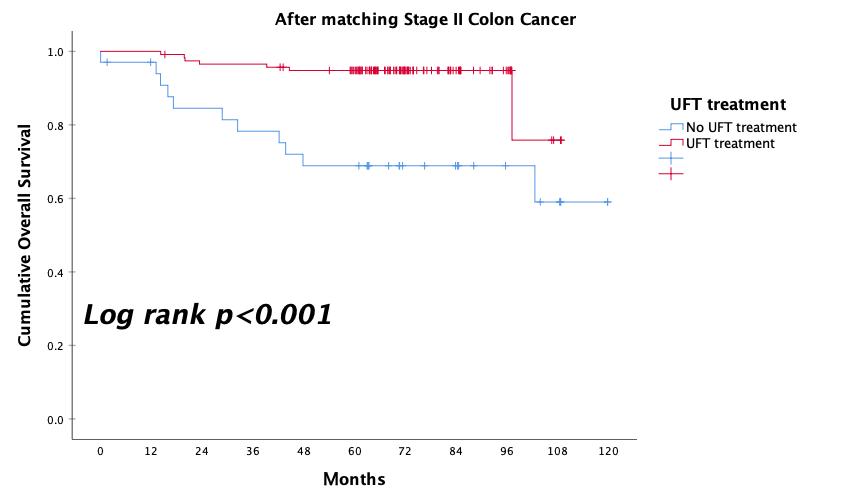


(b) The OS analysis according to UFT treatment in patients with stage II rectal cancer.


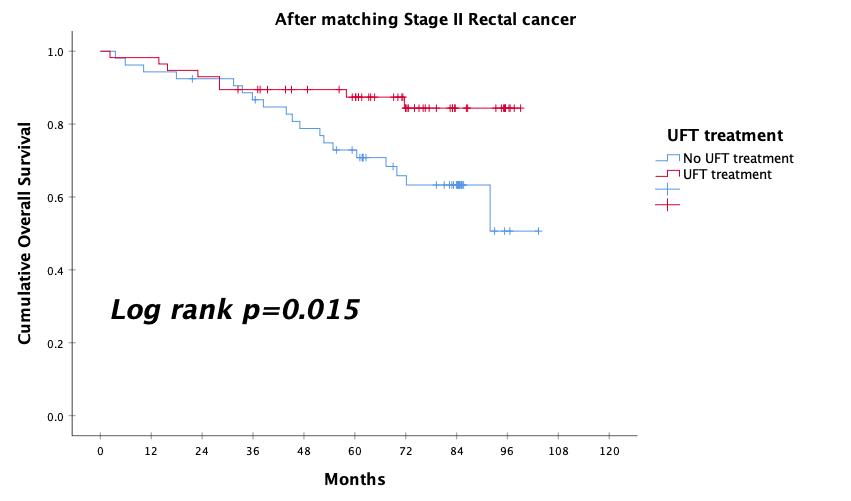

Supplement: Supplementary file 1 — Additional file 1: Supplementary Fig. 1. (a) The OS analysis according to UFT treatment in patients with stage II colon cancer. (b) The OS analysis according to UFT treatment in patients with stage II rectal cancer. [file 12885_2023_11310_MOESM1_ESM.docx]
